# Defining a multifaceted role of *DNAJC5/*CSPα in cell autonomous Alzheimer's disease proteostasis

**DOI:** 10.1002/alz70855_100325

**Published:** 2025-12-23

**Authors:** Matthew J Rosene, Abraham Lopez, Adeeb Ahmad, Skarleth Cardenas Romero, Mei‐Yu Lai, Bruno A. Benitez

**Affiliations:** ^1^ Beth Israel Deaconess Medical Center/Harvard Medical School, Boston, MA, USA; ^2^ Beth Israel Deaconess Medical Center, Boston, MA, USA; ^3^ BIDMC/Harvard Medical School, Boston, MA, USA; ^4^ Beth Israel Deaconess, Harvard Medical School, Boston, MA, USA

## Abstract

**Background:**

*DNAJC5* encodes for cysteine string protein‐α (CSPα), an endolysosomal cochaperone that regulates synaptic integrity, the exocytosis of neurodegenerative‐associated aggregate‐prone proteins, and the autophagy/lysosomal pathway (ALP), a central pathway in Alzheimer's disease (AD). Single‐nuclei RNAseq has shown elevated expression of CSPα in microglia, where its role has not been studied. These findings imply that CSPα may play different roles in brain proteostasis on a cell type‐specific basis. Here, we CSPα‐dependent proteostasis and investigate the role it may play in AD.

**Method:**

Transcriptome and proteomic data was generated from human immortalized neuronal, astrocyte, and microglial lines stably overexpressing wild‐type (WT) and mutant CSPα. Patient‐derived iPSCs carrying mutant CSPα and a CRISPR‐corrected isogenic control were differentiated into cortical neurons and analyzed through immunoblotting and immunostaining. Proteomic (Somascan 7K) data from the frontal, parietal, occipital, and temporal cortex of CSPα mutant carriers (*N* = 4) and healthy controls (*N* = 5) were integrated and analyzed. Biochemical analyses are performed on cell lysates and conditioned media from cells treated with ALP modulators.

**Result:**

Patient‐derived iPSCs from CSPα mutation carriers replicate the formation of CSPα aggregates and autophagic dysfunction found in CSPα mutant brain tissue. Proteomic analysis from human brain tissue and our *in vitro* cell lines reveals altered proteins associated with the ALP. The secretome from the *in vitro* cell lines exhibits altered expression of other cochaperones and proteins associated with inflammation, especially in the microglial cells. Transcriptome from CSPα mutation carriers revealed 35 differentially expressed genes associated with proliferative microglia and 32 genes associated with the interferon/chemokine response. Human cell lines expressing mutant CSPα exhibit high molecular weight aggregates to differing extents across cell types. Neuronal and microglial lines expressing mutant CSPα show increased levels of depalmitoylated CSPα and signs of autophagic flux. Activation of autophagy decreased monomeric and aggregated CSPα, while lysosomal inhibition increased them.

**Conclusion:**

Our study on mutant CSPα reveals cell type‐specific aggregation phenotypes and altered proteostatic pathways, which we believe will have broader implications on AD‐related proteinopathies. Ongoing multi‐omics analysis will further determine cell‐type‐specific CSPα‐associated neurodegenerative pathways, and future functional assays will further elucidate how CSPα regulates AD proteostasis.

